# Occurrence of *β*-Lactamase-Producing Gram-Negative Bacterial Isolates in Water Sources in Cali City, Colombia

**DOI:** 10.1155/2019/1375060

**Published:** 2019-09-08

**Authors:** Monica Vivas Chavez, Luz Dary Caicedo, Jorge Enrique Castillo

**Affiliations:** ^1^Professor Department of Biomedical Sciences, Faculty of Health, Universidad Santiago de Cali, Campus Pampalinda, Calle 5 # 62-00, Cali, Colombia; ^2^Professor Department of Natural Sciences, Exact and Statistics, Faculty of Basic Sciences, Universidad Santiago de Cali, Campus Pampalinda, Calle 5 # 62-00, Cali, Colombia

## Abstract

**Introduction:**

Pollution by domestic, industrial, and hospital wastes of the artificial and natural waters of the city of Cali led us to investigate the presence of Gram-negative bacteria resistant to antibiotics in these aquatic ecosystems.

**Material and methods:**

We used culture-dependent methods and molecular techniques to investigate the prevalence and dynamics of *β*-lactamase producing Gram-negative bacteria in five areas located in channels and rivers that cross the city of Cali in January (dry season) and May (wet season). The association between the variables was determined by the chi-square test, using the statistical package SPSS vs 23.0.

**Results:**

The main species being *Escherichia coli* and *Pseudomonas* spp. with associated resistance to both cefoxitin and cefotaxime were observed in 73.3% isolates during the dry season. Most of the isolates belonged to antibiotype 3 (with resistance to 6 antibiotics), 51.2% in the dry season and 48.9% in the wet season, and they were found especially in the artificial waters of “Intersector Canal (CVC) Sur”.

**Conclusion:**

These results indicate that *β*-lactamase-producing Gram-negative bacteria are widespread in the environment in the aquatic ecosystem of Cali city. The artificial and natural waters that cross the city are finally discharged into the Rio Cauca; this river can then be considered as a medium for the spread of bacterial antibiotic resistance genes.

## 1. Introduction

Antibiotic-resistant bacteria and antibiotic resistance genes (ARGs) which are introduced into aquatic environments greatly affect public health since these environments are used for various purposes [1–5].

Resistant organisms and ARGs in aquatic environments can occur through contamination of these bodies of water with wastes that mainly contain remnants of antibiotics and direct input of antibiotic-resistant bacteria [6, 7]. The major source of antibiotic contamination in surface water comes from effluents from industries, hospitals, and domestic wastewater treatment plants [8–13]. In the case of antibiotic-resistant bacteria, human and animal waste and agricultural activities are the main causes of their introduction into the aquatic environment [1, 4, 6, 8, 13, 14].

ARGs may be horizontally or vertically transferred between communities of environmental bacteria [15–17]. Aquatic environments can act as a facilitator for the exchange of mobile elements responsible for resisting antibiotics.

Different studies reveal the increase of Gram-negative bacteria resistant to antibiotics in all types of surface water, from waste to natural waters of rivers, lakes, and oceans. [18–21].

In Gram-negative bacteria, the presence of *β*-lactamases is an important defense mechanism against the antibiotics of this group [22–24]. The first *β*-lactamases discovered were TEM-1, TEM-2, and SHV-1, later called broad-spectrum *β-*lactamases. The product of mutations of the genes that encode these enzymes gave rise to current extended-spectrum *β*-lactamases (ESBLs), CTX-M family (from chromosomal beta-lactamases of the *Kluyvera* genus), and other less prevalent, all of them included in the functional group 2be of Bush and Jacoby [25]. Since its initial description, more than 300 different ESBLs have been identified, and the majority belongs to the TEM, SHV, and CTX-M families (https://www.ncbi.nlm.nih.gov/pathogens/beta-lactamase-data-resources/). These enzymes are encoded in plasmids and are transferable to other bacteria by conjugation, which has favored their rapid dispersion.

In recent years, the presence of Gram-negative bacilli resistant to carbapenems has been reported due to the production of *β*-lactamases capable of hydrolyzing this group of antimicrobials.

Some publications mention the relevance of four types of enzymes that are becoming important in new clinical isolates and in resistance in Gram-negative bacteria. These are extended-spectrum *β*-lactamases (ESBLs), *β*-lactamases with reduced sensitivity to *β*-lactamase inhibitors, plasmid-mediated *β*-lactamase AmpC, and *β*-lactamases that hydrolyze carbapenems (belonging to the groups C, A and D, and A and B of the Ambler classification (1) or to groups 1, 2 and 2be, and 3a, 3b, and 3c of the Bush classification) [25, 26].

Because *β*-lactamases can be inducible by the antibiotic, the presence of antibiotics in water systems is a potential risk for the generation of resistance in native Gram-negative bacteria [27–30].

In Colombia, the presence of *β*-lactamases in members of the family *Enterobacteriaceae* and nonfermenting Gram-negative bacilli is reported very frequently. Surveillance studies of resistance are carried out mainly in the hospital environment [31–33]. However, there is little information on the identity and distribution of resistance genes in bacterial isolates in other environments. In this study, we report the prevalence of *β*-lactam antibiotic resistance in Gram-negative bacilli isolated from the artificial and natural waters of the city of Cali and the distribution of genetic elements that may be responsible for the observed antibiotic resistance.

## 2. Material and Methods

### 2.1. Study Area and Sampling

The study site is located in Cali city, at coordinates 3° 27′00 ″N 76° 32′00″ W, in southwestern Colombia. It is the third most populated city in Colombia. The main river of the city is the Cauca River, which halfway through the city receives the pollutant load of the so-called “Intersector Canal (CVC) Sur” (sampling site C), which mobilizes the wastewater from the southeast of the city. Following the course of the Cauca River, 11 km downstream is the water treatment plant “Puerto Mallarino” (sampling site D) that supplies 80% of the water consumed by the city. 3.4 km later, there is the wastewater treatment plant “Cañaveralejo PTAR-C” (sampling site A). The city also has the Cañaveralejo and Melendez rivers (sampling site E) that originate in the center of the city and end at the southern CVC Intersector channel.

**Figure 1:**
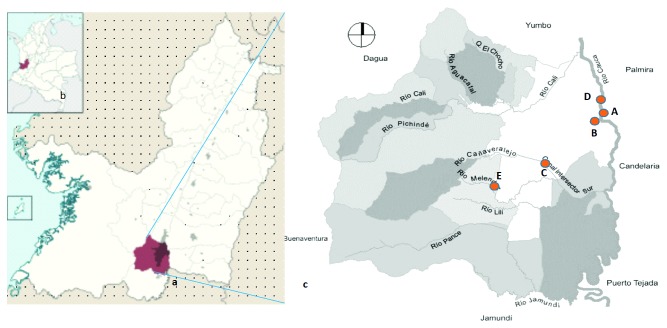
Geographical location of the sampling site. (a) Department of Valle del Cauca, Colombia; (b) City of Cali. (c) Red points show the sites where surface water samples were collected: the wastewater treatment plant “Cañaveralejo PTAR-C” (A), rainwater channel (B) near the wastewater treatment plant, Intersector Canal (CVC) Sur (C), water treatment plant “Puerto Mallarino” (D), and Melendez River (E).

In January (dry season) and May (wet season) of 2017, three samples per location were collected from five areas at the points indicated on the map (Figure 1).

Wastewater effluent and incoming river water samples were collected by point collection in triplicate in 500 ml brown glass bottles protected with an aluminum seal and/or a Teflon cap, completely filled, and kept at 4°C in an icebox containing ice during transportation to the laboratory and processed immediately for the enumeration of bacteria.

### 2.2. Bacterial Counts, Isolation, and Identification

Viable counts in wastewater effluent and river water samples were estimated by preparing serial ten-fold dilutions from 10^–1^ to 10^–6^. A standard volume of each dilution was then inoculated on duplicate plates of CHROMagar total coliforms (CHROMagar™ ECC, DRG International Inc.) to determine coliform counts. After incubation at 37°C for 48 hours, colonies of coliform bacilli were counted.

Water samples were diluted 1 : 1 in 0.85% saline solution prior to inoculation. After overnight incubation, the broth was subcultured onto MacConkey agar (Oxoid Ltd., Hampshire, United Kingdom) to isolate and identify the selected Gram-negative enteric bacteria and cetrimide agar (Merck, Germany) to isolate *Pseudomonas* spp. Bacterial identification was confirmed using the Vitek GNI + card (bioMerieux Vitek Inc., Hazelwood, MO). Pure strains with no more than 24 hours of growth were used and a 1.0 McFarland suspension was prepared in saline solution, which was used to inoculate the Vitek cards at a fixed incubation temperature of 37°C following the manufacturer's instructions. The Vitek system is an automated system that is based on the inoculation of a suspension of microorganisms on cards with certain biochemical reactions panels and allows species-level identification of Gram-negative bacilli.

A total of 80 (31 of *E. coli*, 25 of *Klebsiella* spp., and 24 of *Pseudomonas* spp.) and 86 (42 of *E. coli*, 16 of *Klebsiella* spp., and 28 of *Pseudomonas* spp.) isolates were selected from the water samples in the dry season and wet season, respectively.

### 2.3. Determination of Antibiotic Resistance Profiles

Antibiotic resistance profiles were obtained from 166 isolates. A standardized amount was inoculated (standard 0.5 of McFarland) and antimicrobial susceptibility testing was performed by the disk diffusion method on Mueller–Hinton (MH) agar (Difco Laboratories, Detroit, MI, USA) using interpretative criteria of the Clinical and Laboratory Standard Institute (CLSI) [34]. The following antimicrobials were used: trimethoprim/sulfametoxazol (SXT, 23.75 *μ*g + 1.25 *μ*g), gentamicin (GEN, 10 *μ*g), ciprofloxacin (CIP, 5 *μ*g), ceftazidime (CAZ, 30 *μ*g), cefoxitin (FOX, 30 *μ*g), cefotaxime (CTX, 30 *μ*g), meropenem (MEM, 10 *μ*g), piperacillin/tazobactam (TZP, 40 *μ*g), and cefixime (CFM, 5 *μ*g) (Oxoid Ltd., Hampshire, United Kingdom). ESBL-positive isolates were identified by using the double-disk synergy test with third-generation cephalosporins (cefotaxime, ceftazidime, and cefixime) alone and with clavulanic acid. Quality control was carried out using standard strains of *Escherichia coli* (ATCC 25922) and *Pseudomonas aeruginosa* (ATCC 27953). Intermediate susceptibility to each antibiotic was considered to be resistant.

According to the definitions proposed by Magiorakos et al., resistance to three or more antibiotic classes was defined as multidrug resistant (MDR) [35].

### 2.4. DNA Extraction and Quantification

DNA extraction was carried out from a bacterial culture in LB (Luria–Bertani) broth at 37°C overnight using a genomic DNA extraction kit (MO BIO Laboratories Inc.).

The concentration of DNA in the samples was quantified by using a FOTO/Analyst® Investigator/FX Systems (FOTODYNE Incorporated).

### 2.5. Polymerase Chain Reaction (PCR) to Detect *bla* Genes

PCR assays were used for the detection of *β*-lactam resistance genes: *bla*_TEM_, *bla*_CTX‐M-9_, *bla*_IMP-1_, *bla*_VIM-2_, and *bla*_AmpC_ genes, as shown in Table 1 [31, 36–39].

Amplification was carried out in 50 *μ*l volumes with 5–10 ng (genomic DNA) reaction buffer, 1 U of *Taq* polymerase (Bioline, London, United Kingdom), 200 *μ*M deoxynucleoside triphosphate, 1.5 or 2.5 mM MgCl_2_, 10 pmol of each primer, and 4 *μ*l of DNA as the template. PCR conditions generally were as follows: a hot start at 94°C for 5 min; 35 cycles of either 30 s at 94°C, 45 s at 52°C (*bla*_TEM_-_1_), 45 s at 51°C (*bla*_CXT-M-9_ and *bla*_AmpC_), 1 min at 51°C (*bla*_VIM-2_ and *bla*_IMP-1_), or 60 s at 72°C; and a final step of 10 min at 72°C.

The amplified DNA products were analyzed by conventional 1.5% (wt/vol) agarose gel electrophoresis in 1X TAE buffer and run at 100 V for 1 h. To visualize band migration, the gel was stained with ethidium bromide and observed under UV light. A 100-bp or 1-kb ladder (Gibco BRL, Ontario) was used to estimate the amplicon size.

### 2.6. Statistical Methods

Differences in frequencies of *β*-lactamase genotypes among groups were evaluated using chi-square tests (*χ*2) using contingency tables with a significance level of *p*=0.05. Univariate analysis of variance was performed for inference on differences in the average numbers of multidrug resistance between *β*-lactamase genotypes. Analyses were performed in IBM SPSS Statistics version 23.0 (SPSS Inc., Chicago).

## 3. Results

This study recorded total coliform counts ranging from 2.32 × 10^5^ CFU/ml to 4.57 × 10^5^ CFU/ml in the dry season and 6.6 × 10^3^ CFU/ml to 2 × 10^4^ CFU/ml in the wet season. A variety of organisms such as *Pseudomonas* spp., *Acinetobacter* spp., *Pasteurella* spp., *Aeromonas* spp.*, Proteus* spp.*, Klebsiella* spp., *Enterobacter* spp., *Alcaligenes* spp., and *Escherichia coli* comprised the majority of the isolates.

However, to carry out specific studies on susceptibility to antibiotics and detection of resistance genes, only isolates of *E. coli*, *K. pneumoniae*, and *Pseudomonas* spp. were used, since they are the most abundant, in the amounts indicated in Material and Methods.

### 3.1. Antimicrobial Susceptibility

Isolates were subjected to an antibiotic susceptibility test using 8 different antibiotics from which their antibiotic resistance profiles and multiple antibiotic resistance phenotypes were compiled from the five areas. The results obtained are depicted in Table 2 and revealed that a large proportion of the environmental isolates was resistant to cefoxitin and meropenem (78.8%) during the wet season, followed by ciprofloxacin. During the dry season, resistance to cefoxitin and cefotaxime reached 73.2% followed by resistance to piperacillin/tazobactam (72%).

Ninety-six and a half percentage (96.5%) of the isolates were resistant to >1 antibiotics during the wet season, while 47.7% of the isolates were resistant to 2 or more classes of antibiotics tested during the dry season. The rates of resistance were highest during the wet season (96.6%), and during the dry season, the resistance was lower (75.7%).

All isolates obtained during the dry and wet seasons were grouped into four (1 to 4) different antibiotypes depending on their susceptibilities to 8 different antimicrobial drugs (Table 3). MDR (3 to 8 antibiotics) bacteria were common among the isolates, and these corresponded to the isolates of antibiotypes 1, 2 and 3.

Interestingly, antibiotypes were grouped significantly in the sites tested during the wet season (*p* < 0.05). Most isolates belonged to antibiotype 3 (51.2% in the dry season and 48.9% in the wet season) and were found especially at the site called “Intersector Canal (CVC) Sur”.

In some cases, the isolates were resistant to up to eight different antibiotics and correspond to antibiotype 1; most of these isolates were found at the rainwater channel (23.1% in the dry season and 27.3% in the wet season).

Antibiotype 4 with isolates showed sensitivity to all the antibiotics tested and represented 22% during the dry season but decreased to 3.4% during the wet season (only determined at the site called “Intersector Canal (CVC) Sur”, 10%).

Table 4 shows that the presence of carbapenemases was more common (36.1%) and was detected in 50% of the isolates from the artificial waters of Intersector Canal (CVC) Sur during the wet season, similarly to the ESBL. However, all ESBL isolates showed a lower prevalence (27.1%).

Isolates with AmpC were found in 16.7% and isolates with broad-spectrum *β*-lactamases represented 10.8%, even though they were not detected during the wet season in the channel of rainwater.

### 3.2. *bla* Genes Present in Isolates

We found that 75% and 88.4% of all the isolates identified had at least one type of gene *bla* in the dry and wet seasons, respectively. Of these, 31.3% and 36% were positive for two or more genes, respectively.

The rainwater channel and Intersector Canal (CVC) Sur showed the highest number of isolates with *bla* genes in 75.6% and 40.7%, respectively. These two sites receive artificial water, consisting mainly of domestic and industrial wastewater from the city of Cali, and in the Cauca River, which collects all the artificial and natural waters of the city, the number of isolates with *bla* genes reached up to 24.4% during the wet season.

DNA from a total of 166 isolates was amplified; 82 (49.4%) isolates were identified as containing *bla*_VIM-2_, 75 (45.2%) were identified as containing *bla*_TEM-1_, 38 (22.9%) strains contained *bla*_IMP-1_, 34 (20.5%) strains contained *bla*_AmpC,_ 24 (14.5%) strains contained *bla*_CTXM-9_, and 28 (16.9%) did not amplify (Table 4).

## 4. Discussion

The contamination of artificial and natural surface waters with pathogenic bacteria is a major problem for developed countries and developing countries such as Colombia [40].

We used culture-dependent methods to investigate the prevalence and dynamics of heterotrophic antibiotic-resistant bacteria from natural (water treatment plant “Puerto Mallarino” and Melendez River) and artificial (wastewater treatment plant, rainwater channel, and Intersector Canal (CVC) Sur) water sources. The group of total coliforms comprises a wide range of microorganisms that are found everywhere in aqueous and soil environments; they develop very well in the intestinal tract of humans and mammals (fecal coliforms), as well as in the organic material originated by the decaying vegetation.

Although the natural mortality of coliform populations is very high, especially in fecal coliforms due to adverse conditions in the extraenteric environment, their great abundance could be evidenced during the dry season, reaching orders of 10^5^ CFU/ml, which is in accordance with the reports of McFeters, who reported values of 10^5^–10^10^ CFU/ml of total coliforms in wastewater [41]. Certain environmental conditions (turbidity, temperature, etc.) can keep these populations viable for a long time [42, 43].

However, the amount of the coliform population decreased to an order of 10^3^ CFU/ml during the wet season. The reason for this difference could be the reduction of the flow in the rivers during the dry season, which is maintained mainly by the artificial domestic, hospital, and industrial wastewater which would be the main sources of these bacteria.

Davino et al. [44] and Delgado et al. [45], analyzing the coliform counts in Jatiúca Beach, Brazil, and Bassaseachic Falls National Park, Mexico, respectively, reported that the fecal coliform count was higher during the wet seasons (May, June, and July) than during the dry seasons (November, December, and January), indicating water movement during higher temperatures [44–46].

The presence and persistence of bacteria resistant to antibiotics have been described in surface water, representing a growing public health problem [42–48]. The data collected in the present work allow us to affirm that in the city of Cali, the same problematic situation is presented.

Water samples collected during the sampling period present a pattern of resistance to antibiotics against dispar. The highest number of resistant isolates was obtained significantly to third-generation cephalosporins and *β*-lactamase inhibitors during the dry season, reaching values above 70%, especially in the artificial waters of the rainwater channel and in Intersector Canal (CVC) Sur. The resistance prevalence data of Gram-negative bacilli resistant to *β*-lactams were similar to those detected in the sewage system of the city of Rio de Janeiro [43] and other cities in Brazil [44]. These antibiotics are the most used in the treatment of infections caused by Gram-negative bacilli, which shows the selective pressure that is exerted on bacteria of fecal and natural origin.

In relation to the susceptibility profiles, no statistically significant differences were found between the samples of artificial and natural waters. However, a MDR profile (especially, antibiotype 3) with resistance to more than 6 antibiotics was the most prevalent in the sampling sites. In accordance with the results of the study of Chelosi et al. [48] more than 56% of Gram-negative bacteria from cultured marine sediments in western Mediterranean were found to have resistance to 5 or more antibiotics.

In addition, the percentages of MDR bacteria to antibiotics obtained in this study correspond to the ranges of resistance found by other authors. Thus, MDR isolates from the *Enterobacteriaceae* family have been identified in Bassaseachic Falls National Park, Mexico [46], and in the Almendares River in Cuba [47].

Reports indicate that the frequency of MDR organisms varies according to the environment and period and shows an increasing number of these phenotypes in natural environments [9, 19, 30, 39, 43, 46, 47].

A further objective of this study was to characterize *β*-lactamase-producing Gram-negative bacteria; individual isolates of *β*-lactamase-producing bacteria were found during the dry season (24.4%) and wet season (34.5%). In concordance with these results, the prevalence of Gram-negative bacteria in the aquatic environment has been already reported, especially those of the coliform group. More than 50% of the diseases caused by contaminated water are associated with bacteria from the intestinal microbiota, such as *Enterobacteriaceae* and the coliform group [21, 28, 29]. The contamination of these environments reflects the poor quality, hygiene, and sanitization of the water [1–7].

In this study, we detected carbapenemases, ESBLs, broad-spectrum *β*-lactamases, and inducible chromosomal *β*-lactamases. The largest number of isolates with *β*-lactamases was concentrated in the waters of Intersector Canal (CVC) Sur. This channel collects domestic, hospital, and industrial wastewater from a large part of the city and mixes it with the natural waters of the Melendez and Cañaveralejo rivers to finally be discharged into the Cauca River (the second largest river in Colombia). This situation is repeated in many of the rivers that cross the main Colombian cities. The excessive use of *β*-lactam antibiotics and their inadequate disposition are factors that promote the appearance of resistant organisms in the environment, so their proper use would be an appropriate measure to preserve this powerful family of antibiotics.

Bacteria may inherit resistance to some antibiotics or can develop resistance via spontaneous mutation or the acquisition of resistant genes [14–17]. The resistance in Gram-negative bacteria to *β*-lactam antibiotics is largely due to the acquisition of *bla* genes, which code for a broad group of *β*-lactamases.

We found more than 75% of isolates with *bla* genes during the dry and wet seasons, mainly in artificial waters. *bla*_TEM-1_, *bla*_CTX-M-9_, *bla*_VIM-2_, *bla*_IMP-1_, and *bla*_AmpC_ genes were detected in almost all samples of artificial and natural waters, which shows that these genes are widely distributed in the city's water systems. The finding of this high percentage of *bla* genes in environmental isolates reveals the selective antibiotic pressure that would be exerted on them and/or the dissemination of resistance genes among bacterial populations of water systems.

One limitation of this study was only the identification of the *bla*_CTX-M-9_ gene; although together with the CTX-M-14 enzyme, it is frequently found in Latin American countries, and the identification of CTX-M-15 (CTX-M-1 group) would have been useful because of its wide global distribution [49].

## 5. Conclusions

The study shows that the artificial and natural waters of the city are contaminated with resistant bacteria and can contribute to the spread of these bacteria.

Isolates with resistance to *β*-lactam antibiotics, especially third-generation cephalosporins, were more prevalent during the dry season. However, there was no statistically significant variation from data found in the wet season.

The presence of Gram-negative bacteria resistant to more than 6 antibiotics (represented by antibiotypes 1 and 3) was evidenced in the natural and artificial waters of the city, representing a risk to humans and the environment in that they can act as reservoirs of resistant bacteria and contributing to the storage and diffusion of antibiotic resistance genes.

We also found isolates with resistance to non-*β*-lactam antibiotics showing that these isolates have developed more than one mechanism of resistance. This suggests that these strains have been subjected to multiple selective pressures upon being in the constant presence of antibiotics, thus causing them to become resistant to higher order drugs.

The artificial and natural waters that cross the city of Cali are finally discharged into the Rio Cauca, and this river can then be considered (a) as a medium for the spread of bacterial antibiotic resistance genes, (b) acts as a reservoir for these genes and (c) due to socio-economic pressure, may play a role in the development and evolution of these genes along this river system.

## Figures and Tables

**Table 1:** Primers used in study.

Primer	Oligonucleotide sequence (5′ ⟶ 3)	Size (pb)	Reference
*bla*_TEM-1_	F-5′ATGAGTATTCAACAT TTC CG3′	956	[36]
	R-5′CTG ACA GTT ACC AAT GCT TA3′		

*bla*_*VIM-2*_	F-5′AAAGTTATGCCGCACTCACC3′	865	[37]
	R-5′TGCAACTTCATGTTATGCCG3′		

*bla*_*IMP-1*_	F-5′ATGAGCAAGTTATCCTTATTC3′	741	[38]
	R-5′GCTGCAACGACTTGTTAG3′		

*bla*_CTX-M-9_	F-5′GTGACAAAGAGAGTGCAACGG3′	856	[31]
	R-5′ATGATTCTCGCCGCTGAAGCC-3′		
	R: GCGTTGCCAGTGCTC		

*bla*_AmpC_	F-5′CCC TTT GCT GCG CCC TGC 3′	431	[39]
	R-5′ TGC CGC CTC AAC GCG TGC 3′		

**Table 2:** Distribution of Gram-negative bacteria with resistance to antibiotics at the sampling sites during the dry and wet seasons.

Sampling site												
*Antibiotic*	A		B		C		D		E		Total	
	DS (%)	WS (%)	DS (%)	WS (%)	DS (%)	WS (%)	DS (%)	WS (%)	DS (%)	WS (%)	DS (%)	WS (%)
FOX	26.7	21.7	13.3	18.8^*∗*^	26.7	42^*∗*^	15	4.3	18.3^*∗*^	13	73.2	78.4
CTX	26.7	31.3	13.3	0	26.7	50	15	9.4	18.3^*∗*^	9.0	73.2	36.4
CAZ	22.4	26	16.3	10	24.5	50	20.4	4	16.3^*∗*^	10	59.8	56.8
TZP	28.8	14.6	11.9	3.6	25.4	50	13.6^*∗*^	3.6	20.3	28.6	72	31.8
MEM	26.8	26.1	12.2	15.9	26.8	42	19.5	2.9	14.6	13	50	78.4
STX	28.6	32.4	17.9	11.8	21.4	41.2	17.9	5.9	14.3	8.8	68.3	38.6
CIP	25.6	30.4	16.3	17.9	23.3	37.5	16.3	5.4	18.6^*∗*^	8.9	52.4	63.3
GEN	31.6	26.9	14	11.5	26.3	38.5	14	0	14	23.1	69.5	29.5

Trimetroprim/sulfametoxazol (SXT), gentamicin (GEN), ciprofloxacin (CIP), ceftazidime (CAZ), cefoxitin (FOX), cefotaxime (CTX), meropenem (MEM), piperacillin/tazobactam (TZP); wastewater treatment plant (A), rainwater channel (B), Intersector Canal (CVC) Sur (C), water treatment plant “Puerto Mallarino” (D), and Melendez River (E). DS: dry season, WS: wet season. ^*∗*^*p* < 0.05.

**Table 3:** Susceptibility profile and distribution of antibiotypes of Gram-negative bacteria isolated from artificial and natural waters.

Ant	Sampling site												Profile	
	Isolates		A		B		C		D		E		Resistance	Sensitivity
	DS *n* (%)	WS *n* (%)	DS *n* (%)	WS *n* (%)	DS *n* (%)	WS *n* (%)	DS *n* (%)	WS *n* (%)	DS *n* (%)	WS *n* (%)	DS *n* (%)	WS *n* (%)		
1	10 (12.2)	13 (14.8)	1 (5.3)	—	3 (23.1)	6 (27.3)	—	—	4 (26.7)	3 (60)	2 (14.3)	4 (33.3)	FOX, CTX, CAZ, TZP, MEM, STX, CIP, GEN	—

2	10 (12.2)	29 (33)	3 (15.8)	7 (36.8)	2 (15.4)	12 (54.5)	3 (15.8)	6 (27.3)	1 (6.7)	1 (20)	1 (7.1)	3 (25)	FOX, CTX, STX/CIP	CAZ, TZP, MEM, GEN

3	42 (51.2)	43 (48.9)	8 (42.1)	12 (63.2)	5 (38.5)	4 (18.2)	13 (68.4)	21 (70)	8 (53.3)	1 (20)	8 (57.1)	5 (41.7)	FOX, CTX, CAZ, STX, CIP, TZP/MEM	GEN

4	18 (22)	3 (3.4)	7 (36.8)	—	3 (23.1)	—	3 (15.8)	3 (10)	2 (13.3)	—	3 (21.4)	—	—	FOX, CTX, CAZ, TZP, MEM, STX, CIP, GEN

Total	80	88	19 (23.8)	19 (21.6)	13 (16.3)	22 (25)	19 (23.8)	30 (34.1)	15 (18.8)	5 (5.7)	14 (17.5)	12 (13.6)		

Trimetroprim/sulfametoxazol (SXT), gentamicin (GEN), ciprofloxacin (CIP), ceftazidime (CAZ), cefoxitin (FOX), cefotaxime (CTX), meropenem (MEM), piperacillin/tazobactam (TZP); wastewater treatment plant (A), rainwater channel (B), Intersector Canal (CVC) Sur (C), water treatment plant “Puerto Mallarino” (D), and Melendez River (E). DS: dry season, WS: wet season; Ant: antibiotype.

**Table 4:** Distribution of *β*-lactamase and *bla* genes in Gram-negative bacteria.

*β*-lactamases	Sampling site											
	Total		A		B		C		D		E	
	DS *n* (%)	WS *n* (%)	DS *n* = 19 *n* (%)	WS *n* = 19 *n* (%)	DS *n* = 12 *n* (%)	WS *n* = 22 *n* (%)	DS *n* = 19 n (%)	WS *n* = 30 *n* (%)	DS *n* = 15 *n* (%)	WS *n* = 5 *n* (%)	DS *n* = 15 *n* (%)	WS *n* = 12 *n* (%)
Broad-spectrum *β*-lactamase	1 (1.3)	17 (19.8)	—	3 (3.5)	1 (1.3)	12 (14)	—	—	—	1 (1.2)	—	1 (1.2)
ESBL	14 (17.5)	31 (36)	1 (1.3)	11 (12.8)	2 (2.5)	3 (3.5)	6 (7.5)	15 (50)	4 (5)	1 (1.2)	1 (1.3)	1 (1.2)
AmpC	21 (26.3)	5 (5.8)	7 (8.9)	1 (1.2)	4 (5)	1 (1.2)	4 (5)	—	1 (1.3)	—	5 (6.3)	3 (3.5)
Carbapenemase	36 (45)	24 (27.9)	10 (12.5)	3 (3.5)	3 (3.8)	—	10 (12.5)	15 (50)	7 (8.8)	1 (1.2)	6 (7.5)	5 (5.8)

*bla genes*												
*bla*_TEM-1_	39 (48.8)	36 (41.9)	11 (13.8)	6 (7)	4 (5)	14 (16.3)	9 (11.3)	9 (10.5)	9 (11.3)	4 (2.3)	6 (7.5)	3 (3.5)
*bla*_CTX-M-9_	—	24 (27.9)	—	6 (7)	—	9 (10.5)	—	4 (2.3)	—	3 (3.5)	—	2 (2.3)
*bla*_AmpC_	9 (11.3)	25 (29.1)	1 (1.3)	3 (3.5)	3 (3.5)	10 (11.6)	2 (2.5)	7 (8.1)	—	4 (2.3)	3 (3.8)	1 (1.2)
*bla*_IMP-1_	—	38 (44.2)	—	7 (8.1)	—	17 (19.8)	—	6 (7)	—	5 (5.8)	—	3 (3.5)
*bla*_VIM-2_	47 (58.8)	35 (40.7)	10 (12.5)	4 (2.3)	8 (10)	15 (17.4)	14 (17.5)	9 (10.5)	6 (7.5)	5 (5.8)	9 (11.3)	2 (2.3)

Wastewater treatment plant (A), rainwater channel (B), Canal Sur (C), water treatment plant “Puerto Mallarino” (D), and Melendez River (E). DS: dry season, WS: wet season. *bla*_TEM-1_*p*=0.567^*∗*^ and 0.024^*ψ*^; *bla*_CTXM-9_*p*=0.573^*ψ*^; *bla*_AmpC_*p*=0.189^*∗*^ and 0.008^*ψ*^; *bla*_IMP-1_*p*=0.000^*ψ*^; *bla*_VIMP-2_.

## Data Availability

All the data used in this study are included within the article.
